# Feminization of the Forehead: A Scoping Literature Review and Cohort Study of Transfeminine Patients

**DOI:** 10.1007/s00266-024-04143-6

**Published:** 2024-06-10

**Authors:** Sumun Khetpal, Fadi Dahoud, Aura Elias, Daniel C. Sasson, Erin M. Wolfe, Justine C. Lee

**Affiliations:** 1 Division of Plastic and Reconstructive Surgery, University of California, 200 Medical Plaza, Suite 460, Los Angeles, CA 90095 USA; 2 Division of Plastic and Reconstructive Surgery, Northwestern University, Chicago, IL USA; 3 Division of Plastic and Reconstructive Surgery, University of Southern California, Los Angeles, CA USA

**Keywords:** Forehead feminization, Frontal bone contouring, Hairline advancement, Brow lift, Facial feminization, Gender-affirming surgery

## Abstract

**Background:**

Facial feminization may be performed to alleviate gender dysphoria among transfeminine patients. The upper third of the face has several characteristics, including hairline shape and position, brow position, and forehead protrusion, that may confer feminine identity. The purpose of this study is to conduct a scoping literature review of techniques performed for forehead feminization and to additionally study clinical outcomes within an institutional cohort.

**Methods:**

A systematic literature review was conducted to review articles that discussed techniques and clinical outcomes associated with procedures performed for feminization of the upper third of the face. A retrospective review of patients undergoing such procedures by the senior author was then conducted. Variables collected included demographic factors, operative details, and postoperative outcomes such as complications, revisions, and re-operations.

**Results:**

Initial review yielded sixty-seven articles. Title and abstract review followed by standardized application of inclusion and exclusion criteria resulted in a total of twenty-two studies for analysis. Priorities of forehead feminization entail frontal bossing reduction, frontonasal angle widening, orbital contouring, brow lifting, and hairline advancement. Eighty-five patients were included for analysis. The majority were of Caucasian race (56%) and had type 3 forehead classification (92%). The average planned setback of the anterior table was 4.12 mm.

**Conclusions:**

The core tenets of the feminization of the forehead lie in the overall creation of a harmonic curvature of the forehead with other facial features. Our multi-pronged analysis presents an updated review of these principles, which may help plastic surgeons in performing procedures to feminize the upper third of the face.

**Level of Evidence III:**

This journal requires that authors assign a level of evidence to each article. For a full description of these evidence-based medicine ratings, please refer to Table of Contents or online Instructions to Authors www.springer.com/00266.

## Introduction

Facial feminization surgery (FFS) incorporates a series of procedures ranging from skeletal modification to soft tissue rearrangement in order to address gender dysphoria, defined as incongruence between one’s gender and physical identity [1–3]. The upper third of the face has multiple characteristics, including eyebrow position, degree of frontal bossing, and hairline shape and position, that elicit certain gender cues. Moreover, certain procedures including brow lift, frontal sinus setback, and fat grafting may be performed to create a cis-feminine appearance for the transgender and non-binary populations. Within the literature, such procedures have been proven to confer superior psychosocial outcomes, as well as improve anxiety, affect, sense of meaning, and purpose [4–6].

**Fig. 1:**
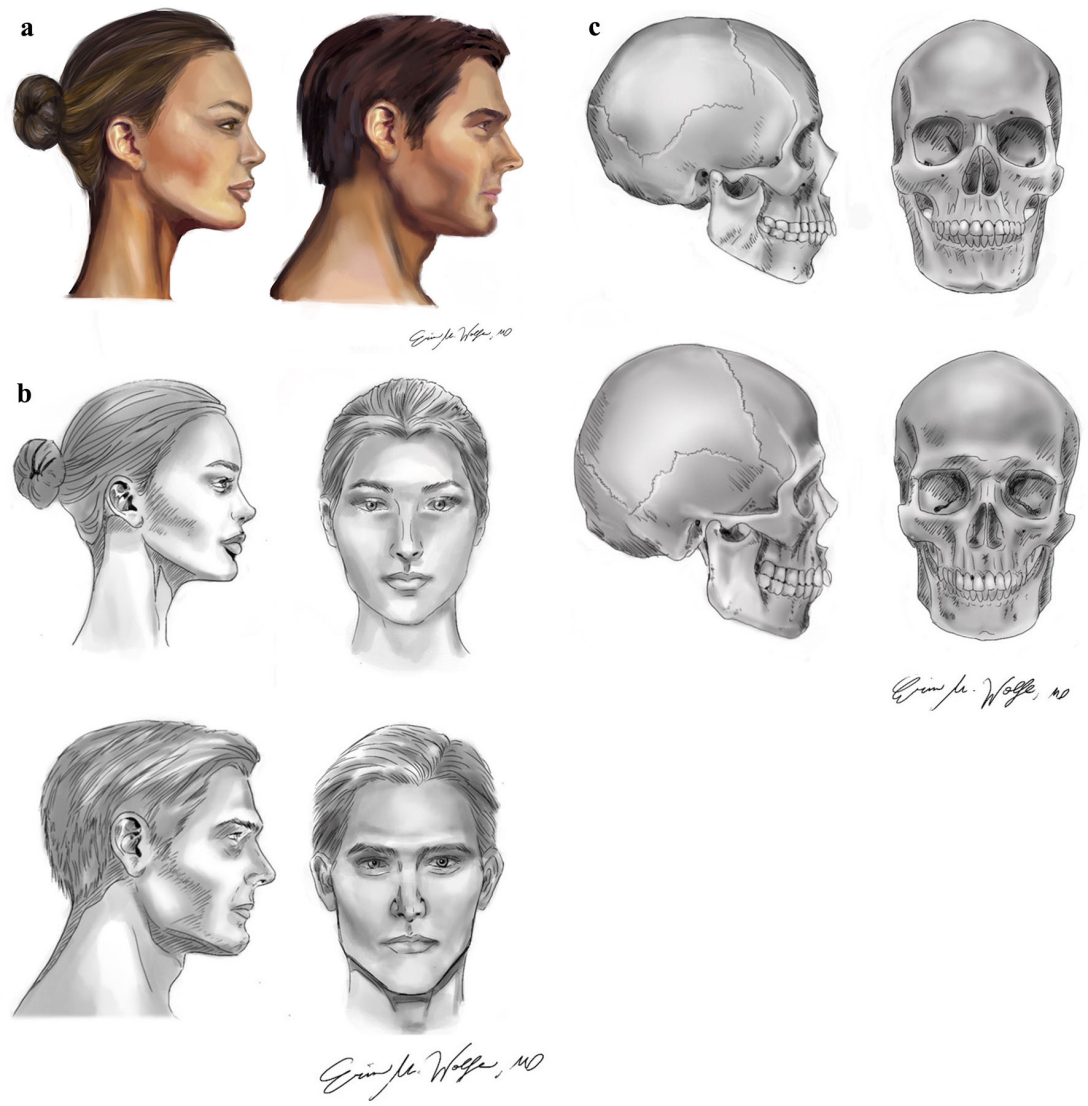
** a** Color illustrations of cis-masculine and cis-feminine facial profiles; **b** Black and white illustrations of cis-masculine and cis-feminine faces; **c** Skeletal differences between male and female skulls

Several features of the upper third of the face have been classically described as binary gender specific [7–10]. The masculine hairline is characterized by an “*M*” shape, the presence of temporal recession, and a long non-hair bearing forehead (6–8 cm); the masculine forehead is sloped with concavity superior to the supraorbital ridge, which has prominent projection; the eyebrows sit at the level of the supraorbital ridge. In contrast, the feminine hairline is defined by an “*O*” shape and short non-hair-bearing forehead (~ 5 cm); the feminine forehead is round and convex with a minimal projection of the supraorbital ridge; the eyebrows are cured, with the lateral limbus peaking superior to the supraorbital ridge. (Figure 1) [11, 12]. The Ousterhout classification has been widely utilized to inform the degree of intervention required for feminization of the forehead; these techniques include frontal burring, osteotomy with frontal sinus setback, augmentation with split calvarial bone graft, and hardware placement [7, 10]. Coronal or pretrichial incisions may be performed in order to adjust forehead height and shape and to perform brow lifts to further refine eyebrow position. With the assistance of a reference female skull, virtual surgical planning (VSP) may be leveraged to promote cis-feminine appearance among patients [13, 14].

While the literature contains general approaches toward feminization of the forehead, brows, and hairline, further analysis is warranted to identify key technical and clinical considerations, as well as to study clinical outcomes when performing feminization of the upper third of the face. Furthermore, the purpose of this study is multi-fold: (1) to review the literature pertaining to the feminization of the forehead, hairline, and eyebrows; (2) to describe operative techniques and clinical considerations aimed at addressing these features, and (3) to assess our institutional approach through a formalized cohort study of patients with clinical outcomes, including operative details and postoperative events.

## Methods

### Literature Review

The literature review was conducted in July 2022 and utilized a combination of the following terms: forehead feminization, forehead feminization surgery, frontal sinus setback, feminizing brow lift, and gender non-conforming forehead feminization. Within the PubMed database, articles were included if they fulfilled the following inclusion criteria: (1) written in the english language; (2) included patients within a formalized cohort or case series; and (3) discussed technical considerations surrounding feminization of the upper third of the face among transfeminine individuals. In order to reduce the possibility of bias, a standardized form was implemented for data collection; variables collected included authors’ names, the title of the article, the year of publication, journal name, number of patients analyzed, as well as technical considerations and recommendations used during facial feminizing surgeries performed by the authors. Articles were categorized by the level of evidence set forth by the American society of plastic surgeons. Any conflicts were resolved through discussion and full-text review among the authors. The search was conducted according to the preferred reporting items for systematic reviews and meta-analyses (PRISMA) guidelines [15–17]. No funding was required to conduct this review of the literature.

### Institutional Clinical Cohort and Clinical Data Extraction

A retrospective cohort study approved by the institutional review board (IRB #11-000925) was conducted among patients who identified as a transgender female, non-binary, or gender non-conforming, and underwent feminization of the forehead, hairline, and/or eyebrow regions. Demographic factors such as ethnicity and insurance status, age at the time of operation, past medical history, and tobacco use were collected. Preoperative assessment was conducted during initial patient consultation and included evaluation of forehead length, forehead classification, lateral hairline recession, sagittal distance from the superior orbital rim to the globe, and degree of temporal recession. Operative details such as type of incision (i.e., coronal, pretrichial, endoscopic), type of frontal setback (i.e., burring only, augmentation of contour concavity superior to frontal bossing with minimal burring, reduction of frontal bossing by osteotomizing and setting back the anterior table of the frontal sinus, augmentation due to severe slope of forehead precluding setback), a specific distance of the planned anterior table setback, and extent of bone grafting were recorded as well. Adjunct facial procedures, complications, and re-operations were additionally recorded for these patients. All analyses complied with the strengthening the reporting of observational studies in epidemiology guidelines.

### Operative Technique

The senior author begins by scoring an incision at the hairline anteriorly and extending to a coronal incision posterolaterally. The posterior scalp is raised in the subgaleal plane to the occiput, and the anterior scalp flap is advanced from the galeal to the pericranium. Laterally, the dissection proceeds immediately over the deep temporal fascia taking care to stay well below the frontal branch of the facial nerve. The orbits are dissected superiorly to the level of zygomaticofrontal suture. Using a virtually planned three-dimensional (3D) guide, the anterior table of the frontal sinus osteotomized the anterior table of the frontal sinus to the frontonasal junction.

Next, the virtually planned, custom forehead reshaping guide was placed on the frontal bone and secured; the frontal bone is drilled at varying depths based on the guide. A pineapple burr was then used to burr down the entire forehead, superior orbital rims, lateral orbital rims, as well as the radix to ensure a smooth transition. After reshaping, the anterior table piece was replaced and secured with a straight plate. For many patients, a split cranial bone graft is harvested and used to reconstruct the gaps of the anterior table of the frontal sinus. Bone dust may be used to blunt the transitions between the bones of the forehead and then the parietal cranial bone graft donor site***.*** The non-hair-bearing skin of the forehead is marked out for the advancement of the scalp and, laterally, for the brow lift. Bone channels may also be created. The non-hair-bearing forehead is then decreased. The scalp is then closed in a typical fashion.

## Results

### Literature Review

**Fig. 2:**
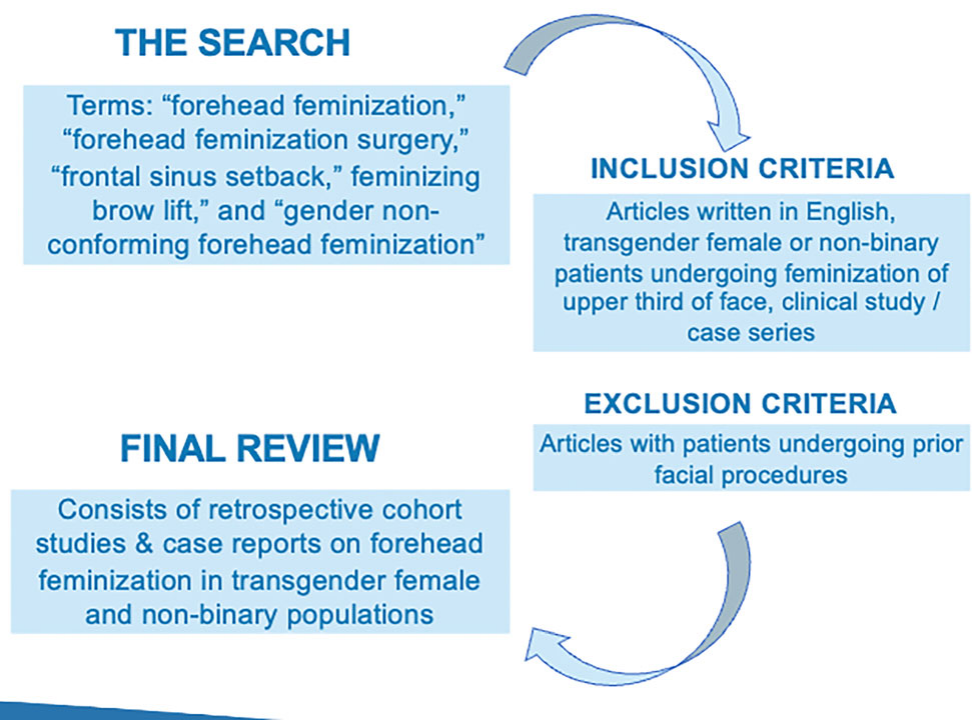
Literature search flow diagram

Initial review yielded sixty-seven articles. The title and abstract review, followed by a standardized application of inclusion and exclusion criteria, resulted in twenty-two studies for analysis. A flow diagram that details this process of isolating articles can be viewed in Fig. 2. These studies included a diverse cohort of authors, publications, and years of publication (Table 1) [18–37].

**Table 1:** Scoping literature review of forehead feminization in transgender female and non-binary populations

First author	Title	Year	Journal	Patient Sample	Technical Considerations and Recommendations	Level of evidence
Ousterhout, DK.	Feminization of the forehead: contour changing to improve female aesthetics	1987	Plastic and Reconstructive Surgery	26	•Group 1: correct with bone reduction •Group 2: contour the frontal bone, augment the concavity above the frontal bossing •Group 3: frontal sinus osteotomy, anterior sinus wall and supraorbital rim set back; contour lateral and superior bone of the sinus and orbital rim	IV
Dempf, R.	Contouring the forehead and rhinoplasty in the feminization of the face in male-to-female transsexuals	2010	Journal of Cranio-Maxillo-Facial Surgery	1	•Reduction of frontal bossing: subperiosteal coronal incision, anterior wall mobilized and stabilized with resorbable osteosynthesis plates. •Widening of nasofrontal angle: from 110° to 130°	V
Hoeing, J.	Frontal bone remodeling for gender reassignment of the male forehead: a gender-reassignment surgery	2011	Aesthetic Plastic Surgery	21	•Hydroxy- apatite cement (HAC) used as an alternative for the correction of cranial vault irregularities	IV
Spiegel, JH.	Facial determinants of female gender and feminizing forehead cranioplasty	2011	The Laryngoscope	100/168	•Burring technique alone: ideal for patients with a small frontal sinus, relatively posterior, and covered with a thick anterior table bone •Island technique: create ‘‘islands’’ of bone over the frontal sinus and set them back in interlocking fashion without violating frontal sinus •Osteotomy and set-back technique: burring; osteoplastic flap created, reduced, and secured into opening of forehead	I
Altman, K.	Facial feminization surgery: current state of the art	2012	International Journal of Oral and Maxillofacial Surgery	N/A	•Trichophytic incision following the hairline: for hairline modification and encouraging hair growth •Increase dimension of anterior orbital rim: all patients should undergo orbital rim contouring	V
Capitán, L. et al.	Facial feminization surgery: The Forehead. Surgical Techniques and analysis of results	2014	Plastic and Reconstructive Surgery	172	•Modified coronal approach: provides excellent access to frontonasal-orbital region and easier to reposition soft tissues •Repositioning technique (Osteotomy and Osteosynthesis): allows for modification of the nasoglabellar angle •Oblique cutaneous incision with a 35° - 45 ° scalpel inclination	IV
Capitán, L. et al.	Facial feminization surgery: simultaneous Hair Transplant during Forehead Reconstruction	2017	Plastic and Reconstructive Surgery	65/492	•Specific HLS recommendations based on transgender female hairline types •Modified temporoparietal (anterior coronal) approach: patients with good hair density and an absence of hair miniaturization •Modified temporoparietooccipital (posterior coronal) approach: patients with low temporoparietal hair density and temporoparietal hair miniaturization	IV
Altman, K.	Forehead reduction and orbital contouring in facial feminization surgery for transgender females	2018	Journal of Oral and Maxillofacial Surgery	N/A	•Group I: burring with pear bur •Group II: forehead reduction with bur; may need filling for potential concavity of superior forehead •Group III: contour forehead and osteotomy of the anterior table •Bony plate cut with Toller fissure at 45° angle, trimmed with pear burr, inferior aspect placed into a retruded position under a pericranial bridge	V
Villepelet, A. et al.	Fronto-orbital feminization technique. A surgical strategy using fronto-orbital burring with or without eggshell technique to optimize the risk/benefit ratio	2018	European Annals of Otorhinolaryngology, Head and Neck Diseases	8	•Frontal remodeling: •Simple burring with acrylic then diamond burr: used for small frontal sinus with thicker anterior walls •Eggshell burring technique: used for highly pneumatized sinus with a thin anterior wall and groups II-III •Lateral canthopexy: stretch and raise the palpebral sling	IV
Capitán, L. et al.	The upper third in facial gender confirmation surgery: forehead and hairline	2019	Journal of Craniofacial Surgery	129	•If rhinoplasty involved: a conical burr is used to lower the frontonasal transition to the desired position •Piezoelectric scalpel for osteotomy: to free the supraorbital nerve or if patient has a thick bony septum	IV
Di Maggio, M.	Forehead and orbital rim remodeling	2019	Facial Plastic Surgery Clinics of North America	N/A	•Radical reshaping of orbit: ostectomy of superolateral orbital rim •Increase orbital height: bur beneath the upper border of the orbital aperture •Selective Sinus Ablation: •Type A anatomy (Frontonasal duct open and functional): sinus mucosa removed, and sinus walls burred out •Type B anatomy (Sinus is defunctionalized): burr out mucosa and ablate the sinus with bone chips and pasting •Hair implant recommended as separate procedure to get better results	V
Eggerstedt, M.	Setbacks in forehead feminization cranioplasty: a systematic review of complications and patient-reported outcomes	2020	Aesthetic Plastic Surgery	673	•Complications are rare: 1.3% and need for revision is low: 0.4% •Complication types: Transient CSF leak, transient frontal branch weakness, transient chemosis, pneumosinus dilatans, recurrent fluid collection	III
Eisemann, BS. et al.	Technical Pearls in frontal and periorbital bone contouring in gender-affirmation surgery	2020	Plastic and Reconstructive Surgery	N/A	•Anterior table osteotomy: use reciprocating saw with blade at 45-degree angle •Anterior table contouring: Complete thinning of ipsilateral side before contouring contralateral side •Stabilizing the anterior table during contouring: use finger only to secure bone, do not use instrument to stabilize.	V
Garcia-Rodriguez, L. et al.	Scalp advancement for transgender women: Closing the gap	2020	The Laryngoscope	29	•Author’s technique (combination of brow lift, frontal cranioplasty, & scalp advancement) both advances and rounds the hairline •overall scalp advancement of 2.01 cm	IV
Spiegel, JH.	Gender affirming and aesthetic cranioplasty: what's new?	2020	Current Opinion in Otolaryngology & Head and Neck Surgery	N/A	•Patients should be on feminizing hormones to stabilize hair follicles and prevent ‘male pattern baldness’ •Use of titanium microplates for fixation of bone: faster fixation and a smoother contour. •3D-cutting guides can be helpful for novice surgeons but can provide a false sense of security	V
Telang, PS.	Facial feminization surgery: a review of 220 consecutive patients	2020	Indian Journal of Plastic Surgery	220	•Hairline incision: addresses receding/ M pattern hairline while providing lifting effect to eyebrows •Recommends a top-to-down and two-stage surgical approach for smoother recovery •Remove portion of the outer table of frontal sinus and cover it with a titanium mesh and pericranial flap	IV
Basa, K.	Frontal bone cranioplasty for facial feminization: long-term follow-up of postoperative sinonasal symptoms.	2021	Facial Plastic Surgery and Aesthetic Medicine	98	•No difference in SNOT scores, or sinus and headache symptoms in patients with violation of the frontal sinus compared to those without •SNOT scores did not vary between this cohort and the non-symptomatic control group population	IV
Dang, B. et al.	Evaluation and treatment of facial feminization surgery: part 1. forehead, orbits, eyebrows, eyes, and nose	2021	Archives of Plastic Surgery	N/A	•Hairline advancement: •Reduction of the length of the non-hair bearing forehead: pretrichial coronal incision •Galeotomies and intraoperative tissue expansion for further advancement •Simultaneous hair transplantation with forehead reconstruction •Temporal fossa augmentations: hyaluronic acid filler, autologous fat or implants	V
Hohman, MH. et al.	3D-Printed custom cutting guides facilitate frontal cranioplasty in gender affirmation surgery	2021	Journal of Craniofacial Surgery	5	•Notable better definition of the frontal sinus border compared to transillumination with an endoscope •Can be used for removal of the anterior table for sinus obliteration or oncologic resection	V
Louis, M. et al.	Narrative review of facial gender surgery: approaches and techniques for the frontal sinus and upper third of the face	*2021*	Annals of Translational Medicine	N/A	Hairline approaches: •Non-surgical: estrogen or hormone replacement therapy •Surgical: FUT, FUE, and HLS Role of VSP: location of frontal sinus can be considered preoperatively; frontal sinus setback faster and more accurate	IV
Pansritum, K.	Forehead and hairline surgery for gender affirmation	2021	Plastic and Reconstructive Surgery	23	Grid method in combination with clinical classification (Ousterhout’s) is the best method to achieve facial feminization •provides common reference points between x-ray and frontal bone intraoperatively and allows surgeons to do a 1-piece frontal bone osteotomy Frontal sinus is the key factor •Height of the frontal sinus highly determines the plan for osteotomy •Use height as only parameter because it is related to the width Fronto-orbital reassembly: primary bone contact and maintenance of a reduced bone gap •Sutures or small wires used instead of fixation with plates and screws; Bone powder rarely used	IV
Tawa, P. et al.	Three-dimensional custom-made surgical guides in facial feminization surgery: prospective study on safety and accuracy	2021	Aesthetic Surgery Journal	45	•3D custom made surgical cutting guides have a 90.8% accuracy rate on forehead procedures on type III foreheads	IV

*HAC*, hydroxy- apatite cement; *HLS*, hairline lowering surgery; *SHT*, simultaneous hair transplant; *DHT*, deferred hair transplant; *SNOT*, postoperative sinonasal outcome Test; *FUT*, follicular unit transplantation; *FUE*, follicular unit extraction; *VSP*, virtual surgical planning

### Retrospective Chart Review

Single-surgeon retrospective chart review yielded eighty-five patients meeting the inclusion criteria. Moreover, such criteria included patients who identified as transgender female, non-binary, or gender non-conforming, and additionally, underwent feminization of the forehead, hairline, and/or eyebrow regions. The majority of patients were of Caucasian race (56%), and between 10–29 years of age at the time of surgery (53%) (Table 2). Type 3 forehead classification was the most common (92%). Most patients exhibited lateral hairline recession (79%) and decreased soft tissue volume of the temple regions (61%).

**Table 2:** Demographics of forehead feminization patients

Variable	*n* (%)
Gender identity	
Female	79 (93%)
Non-binary	5 (6%)
Agender	1 (1%)
Age	
Mean (SD) (years)	32.4 (9.9_
Ethnicity	
Caucasian	48 (56%)
Hispanic/latino	20 (24%)
Asian	6 (7%)
African American	4 (5%)
Other	7 (8%)
Smoker status	
Former smoker	17 (20%)
Never smoker	68 (80%)
Forehead classification	
Type 1	7 (8%)
Type 3	77 (92%)
Lateral hairline recession	
Yes	68 (80)
No	17 (20)
Decreased soft tissue volume of the temple regions	
Yes	60 (71%)
No	25 (29%)
Medical history	
DM	3 (4%)
HTN	2 (2%)

For the majority of patients, the incision was scored anteriorly along the hairline and extended into a coronal incision posterolaterally. The majority of patients underwent a reduction of frontal bossing and the frontonasal angle by osteotomy and anterior table setback (92%) with an average planned setback of the anterior table was 4.12 mm (Table 3). Pineapple burr was used for recontouring of the forehead on nearly all patients (99%). Bone grafting under the temporalis muscle and bilateral corrugator resections were only done in (1.1%) and (3.5%) of the patients, respectively. The average follow-up was 12.1 months. Mucocele was detected in one patient (1%) requiring anterior table reconstruction with split calvarial bone graft.

**Table 3:** Operative details

Operative	*n* (%)
Type of incision	
Hairline	74 (87%)
Coronal	11 (13%)
Brow Lift	
Temporal	53 (62%)
Bone channels	29 (34%)
Coronal	3 (4%)
Frontal sinus reconstruction/fixation	
Autologous bone graft (split calvarial)	78 (92%)
Titanium mesh	2 (2%)
Metal plates	76 (90%)
Average anterior table setback	4.12 mm
Complications	
Mucocele	1 (1%)
Re-operations/revisions	
Brow lift revision	5 (6%)
Scar revision	5 (6%)
Bony recontouring	1 (1%)
Removal of hardware	1 (1%)
Mean follow-up period	12.1 Months, Range: 3 weeks–3 years

**Fig. 3:**
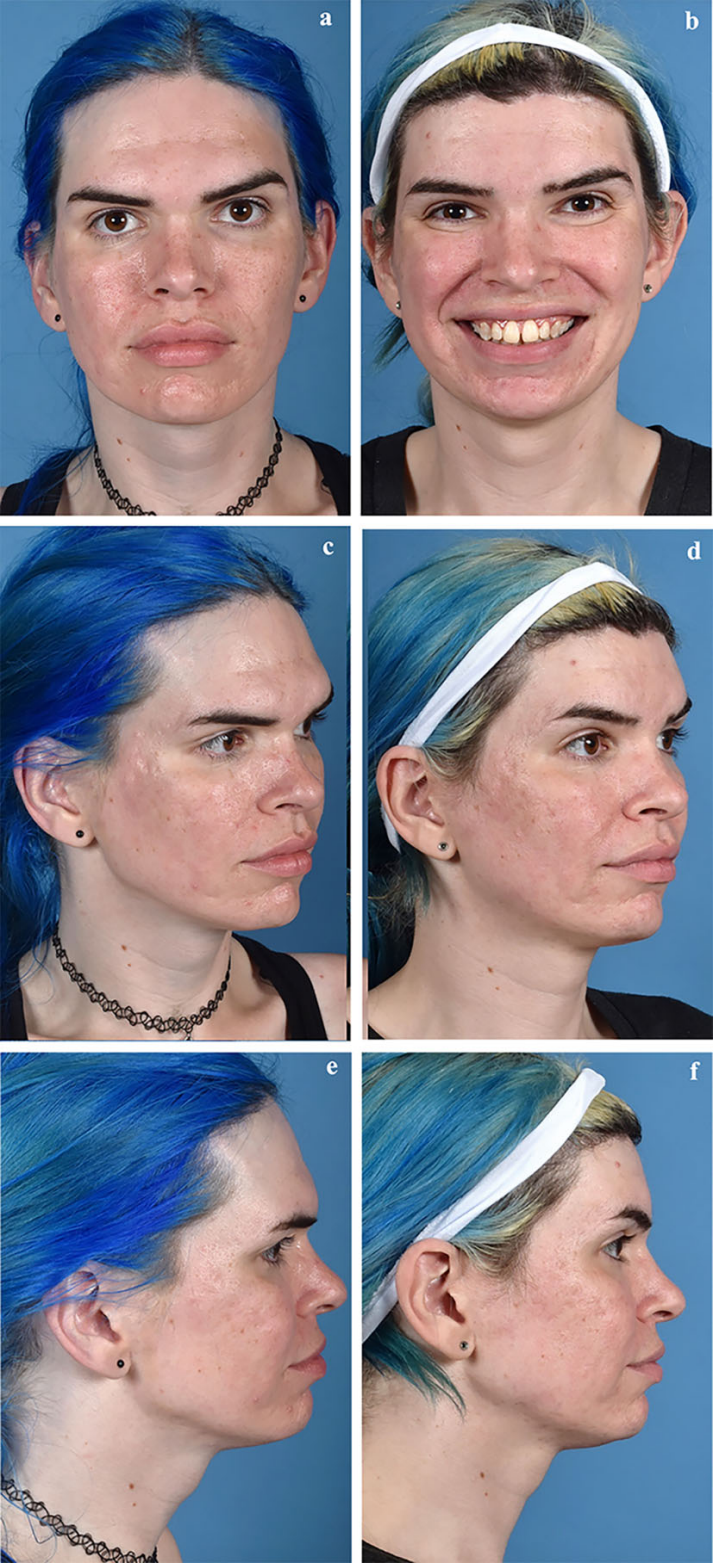
Case example. 28-year-old female (assigned male at birth) who underwent hairline incision for advancement of hairline, anterior table of the frontal sinus setback (6 mm), and brow lift with bone channels. Left panels depict preoperative photographs, right panels depict photographs 10 months postoperative. **a**, **c**, and **e** denote pre-operative facial photographs; **b**, **d**, and **f** correspond to post-operative facial photographs. Copyright retained by senior author

**Fig. 4:**
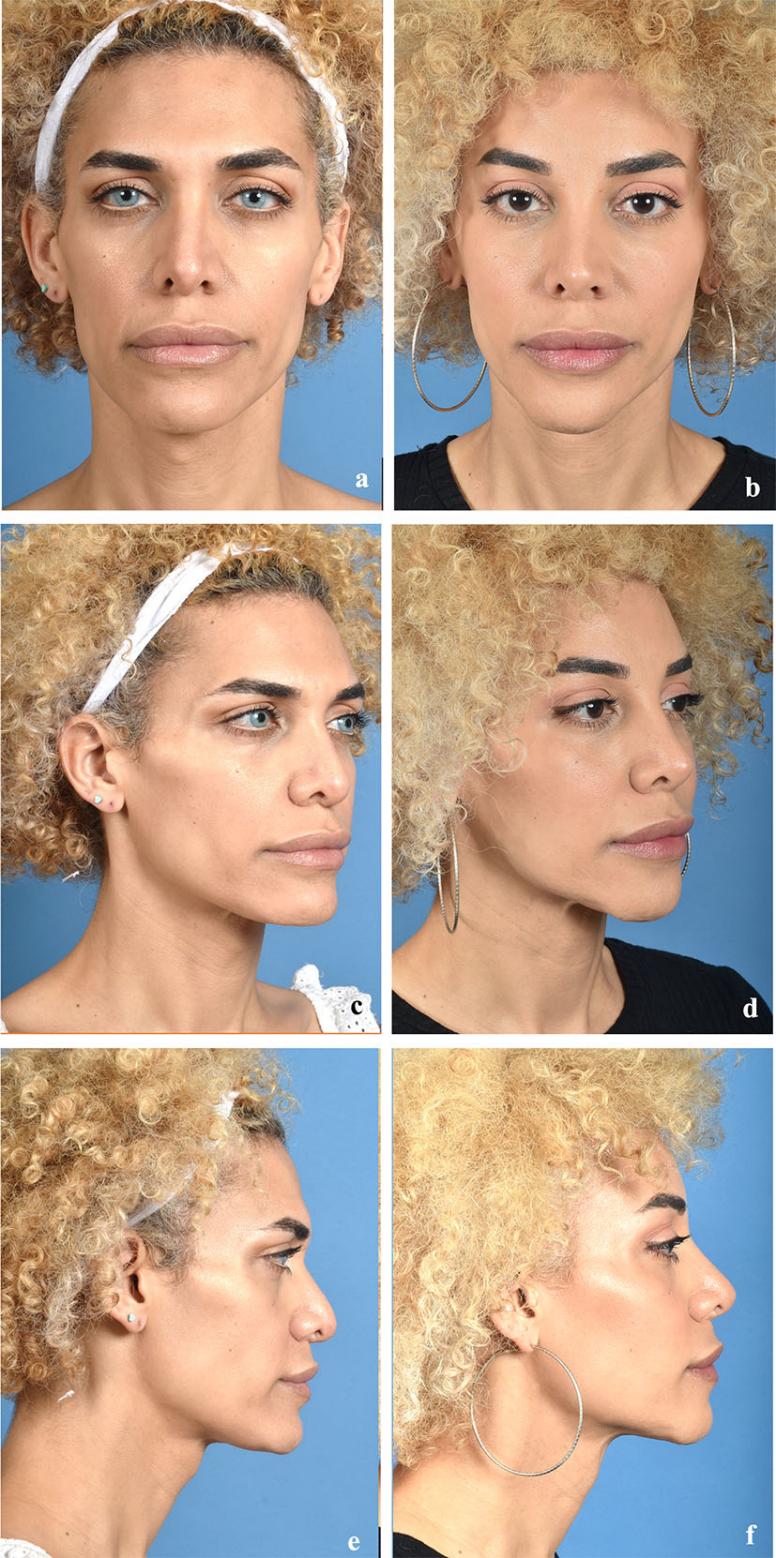
Case example. Forty-seven-year-old female (assigned male at birth) who underwent hairline incision for advancement of hairline, anterior table of the frontal sinus setback (5 mm). Left panels depict preoperative photographs, right panels depict photographs 18 months postoperative. **a**, **c**, and **e** denote pre-operative facial photographs; **b**, **d**, and **f** correspond to post-operative facial photographs. Copyright retained by senior author

A case example (Fig. 3) illustrates the combination of multiple techniques utilized for feminization of the upper third of the face. Moreover, this 28-year-old female (assigned male at birth) patient underwent hairline incision for hairline advancement, anterior table of the frontal sinus setback (6 mm), and brow lift with bone channels. We present an additional case example (Fig. 4) that exhibits forehead contouring via anterior table of frontal sinus setback for a 47-year-old female (assigned male at birth) undergoing facial feminization.

## Discussion

Recontouring and reconstruction of the forehead is an important aspect of facial feminization. Modifications of the bony contours of the forehead such as the orbital rims and frontal bossing are sought after in order to reduce the masculine characteristic in both natal female and transfeminine patients. This study sought to review the literature on facial feminization in transgender women and non-binary patients describing techniques that address features commonly seen in this cohort, as well as a robust review of our institutional experience with this population.

### Technical Considerations

#### Forehead Reduction

Major differences in masculine and feminine forehead anatomy are forehead inclination, frontal bossing, and cranium thickness [8]. In the profile view of the masculine forehead, it is noticeable that it tends to slope more posteriorly compared to the more vertical feminine forehead. The feminine forehead slope has an average posterior inclination of 5.9°compared to the masculine 9.8°. The masculine forehead tends to have an excessive projection of the supraorbital ridge (glabellar region) and a more prominent superciliary arch, creating an appearance of excessive frontal bossing when paired with the masculine straight, posterior forehead inclination. In contrast, the feminine skull has a more prominent superior forehead, providing a more harmonic, rounded shape. The masculine cranium is also thicker and has a larger frontal sinus, contributing to its frontal bossing [7, 8, 10].

To achieve a more feminine appearance, techniques must be performed in order to reduce supraorbital projection, ultimately creating a more continuous, curved contour. As observed in our literature review, there are several techniques to accomplish this reduction for different forehead classification types; these include reduction of frontal bossing, island setback, and osteoplasty with plating [7, 10]. Our cohort consisted predominantly of patients with type 3 foreheads (92%), meaning the ideal bone reduction could not be achieved by contouring alone due to excessive anterior projection of the supraorbital rim. Our protocol is similar to those outlined in this review, including the typical frontal sinus osteotomy in addition to the bony contouring of the lateral and superior orbital rim.

One of the primary unique characteristics in our cohort is the reconstruction of all forehead reconstruction with autologous bone within minimal usage of titanium plates. For all type III forehead reconstructions, split parietal calvarial bone was harvested to reconstruct all of the bony gaps from the osteotomies. While the understanding of the long-term outcomes cannot be compared to usage of titanium mesh, bone cement, or incomplete reconstruction at this point in time, no patients in our institutional cohort required revision of the frontal bone reconstruction for contour abnormalities, bone graft infection, or hardware infection.

The disproportionate prominence of the superolateral orbital ridge provides an angular and square shape in the masculine face. Furthermore, the glabellar region and the superciliary arches are more pronounced, causing a deep-set appearance of the eyes. Sagittal orbital length, measured from posterior to the superior orbital ridge, tends to be less than 10 mm in masculine faces [7, 10]. To achieve a more feminine, rounded orbital rim, contouring the outer third of the superior orbital rim may increase the dimensions of the anterior orbital rim and thus, decrease ridge projection.

#### Frontonasal Angle Burring

The frontonasal angle, which is created by the intersection of the glabella-nasion line and the nasal dorsum, is more obtuse (134°) in a feminine forehead compared to the that of a masculine forehead (120°) [8]. In forehead feminization surgery, a primary goal is to widen the frontonasal angle, achieved by burring the supra-glabellar area as previously described. In addition to the supra-glabellar burring, several studies by Osterhout have suggested further revision during rhinoplasty to lower the frontonasal transition to a more ideal location [7, 10]. Moreover, these studies have concluded that the combination of frontal bone reduction and rhinoplasty can not only provide superior control of the frontonasal angle. In our cohort, rhinoplasty was performed in all patients and provided additional feminization to the face. While our study did not objectively assess the change in nasofrontal angle, future studies could utilize technologies such as Vectra to better quantify these changes.

#### Hairline Advancement and Temporal Recession Reduction

Masculine hairlines tend to have an M shape due to lateral recession, while feminine hairlines are more rounded. In addition, they have a larger, non-hair-bearing forehead (6–8 cm) compared to the feminine forehead (5 cm) [11, 12]. To advance the hairline, decrease the length of the non-hair-bearing forehead, and address lateral recession, most surgeons prefer a hairline incision although hair transplantation or a combination of both techniques are possible. If a hairline correction is not necessary, a coronal approach is performed.

#### Temporal Augmentation

In evaluating soft tissue differences, masculine foreheads have greater temporal hollowing. Many substances have been considered to augment the temporal fossa, including porous polyethylene, autologous fat, fillers, implants, and autogenous bone grafts. Hydroxyapatite cement has been well-described for correction of temporal hollowing and overall cranial irregularitiesl; however, the risk of infection should be carefully considered. In our cohort, an autogenous bone graft under the temporalis muscle was used in one patient (1%); however, the remainder of patients received augmentation using autologous fat grafting.

#### Brow Lift

It is important to ensure the eyebrows are properly positioned following the remodeling of the orbital ridge. Masculine eyebrows rest at the level of the supraorbital ridge, while feminine eyebrows rest above the supraorbital ridge and display a more arched shape. A simultaneous brow lift can raise and arch the eyebrows into a more feminine position and shape. Both coronal and pretrichial incisions provide control when repositioning the eyebrows atop newly sculpted bone.

### Postoperative Procedures and Complications

Within our cohort of eighty-five patients, one patient experienced a mucocele due to narrowing of the superior aspect of the sinus with resultant trapping of sinus mucosa. Of note, the nasofrontal outflow tract was intact and drainage inferior to the trapped mucosa was normal. The patient required reoperation to readvance the frontal bone superiorly and has recovered without sequelae a year following reoperation. While rare in the short-term follow-up, future studies assessing the long-term effects of frontal sinus setback and the role of sinus functionalization are fundamental in preventing such complications. Fifteen percent of patients required additional operations after their initial forehead feminization. These revisions included brow lift revision (6%), scar revision (6%), bony recontouring (1%), and removal of hardware (1%).

### Limitations

There are several limitations in this study that warrant consideration. First, there is inherent variability in the facial contouring techniques performed by surgeons within the analyzed studies. Second, the variability in the primary outcomes measured across different studies made it challenging to directly compare and contrast differing approaches by surgeons. Third, our institutional cohort largely comprised of transgender females who underwent facial feminizing procedures in accordance with cis-normative feminine esthetic ideals. While a small sample of non-binary patients was included, our study did not explore the unique technical and esthetic considerations surrounding the feminization of the upper third of the face in this population. Fourth, our analyzed sample was homogenous in terms of ethnicity, and thus, did not allow an opportunity to explore how feminizing rhinoplasty can preserve key physical traits associated with specific ethnicities.

## Conclusions

This study presents a scoping literature review of gender-affirming forehead feminization, as well as a retrospective review of the senior author’s experience. The primary principles of feminization of the forehead include: (1) feminization of the frontal bones and orbital rims, (2) advancement and feminization of the hairline with reduction of the temporal recession, (3) increased volume in the temporal hollow lateral to the forehead, and (4) elevation and feminization of the eyebrows. Nonetheless, while the goals may be similar for each case, an individualized approach must be commissioned to provide an adequate modification that best aligns a patient’s forehead appearance with their desired gender identity.
